# Unexpected Coexisting Myocardial Infarction Detected by Delayed Enhancement MRI

**DOI:** 10.1155/2009/370542

**Published:** 2009-08-25

**Authors:** Edouard Gerbaud, Henri De Clermont-Galleran, Matthew Erickson, Pierre Coste, Michel Montaudon

**Affiliations:** ^1^Service des Soins Intensifs Cardiologiques, Hôpital Cardiologique du Haut Lévêque, CHU de Bordeaux, Avenue de Magellan, 33604 Pessac Cedex, France; ^2^Service de Médecine Nucléaire, Hôpital Cardiologique du Haut Lévêque, CHU de Bordeaux, Avenue de Magellan, 33604 Pessac Cedex, France; ^3^Unité d’Imagerie Thoracique et Cardiovasculaire, Hôpital Cardiologique du Haut Lévêque, CHU de Bordeaux, Avenue de Magellan, 33604 Pessac Cedex, France

## Abstract

We report a case of an unexpected coexisting anterior myocardial infarction detected by delayed enhancement MRI in a 41-year-old man following a presentation with a first episode of chest pain during inferior acute myocardial infarction. This second necrotic area was not initially suspected because there were no ECG changes in the anterior leads and the left descending coronary artery did not present any significant stenoses on emergency coronary angiography. Unrecognised myocardial infarction may carry important prognostic implications. CMR is currently the best imaging technique to detect unexpected infarcts.

## 1. Introduction

Myocardial infarcts are routinely detected by single photon emission computed tomography (SPECT) myocardial perfusion or CMR imaging. Rioufol et al. showed with intravascular coronary ultrasound (IVUS) that although only one lesion is clinically responsible during an acute coronary syndrome (ACS), ACS seems to be associated with pan-coronary destabilisation [1]. CMR is the gold standard technique to visualise myocardial necrosis and evaluate myocardial viability after an acute coronary syndrome [2]. Moreover, Wagner et al. demonstrated that although SPECT and CMR detect transmural myocardial infarcts at similar rates in animals, CMR systematically detects subendocardial infarcts that are missed by SPECT [3]. We present a case of an unexpected coexisting myocardial infarction (absence of significative stenosis on coronary angiography) detected by delayed enhancement MRI, which was unseen by SPECT.

## 2. Case Report

**Figure 1:**
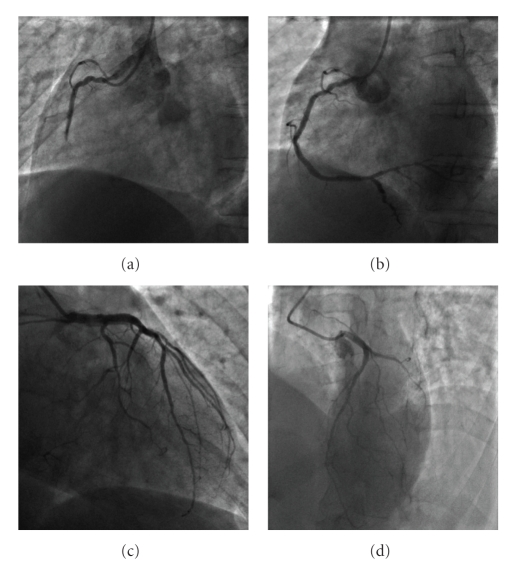
Initially, the coronary angiography showed an acute thrombotic occlusion on the second segment of the right coronary artery (Panel (a)). The patient underwent angioplasty and stenting with a final good result (Panel (b)). Coronary angiography revealed a severe stenosis on a minor circumflex coronary artery (Panel (c)). There were many diffuse lesions on the left anterior descending coronary artery and his branches without significant stenosis (Panel (d)).

**Figure 2:**
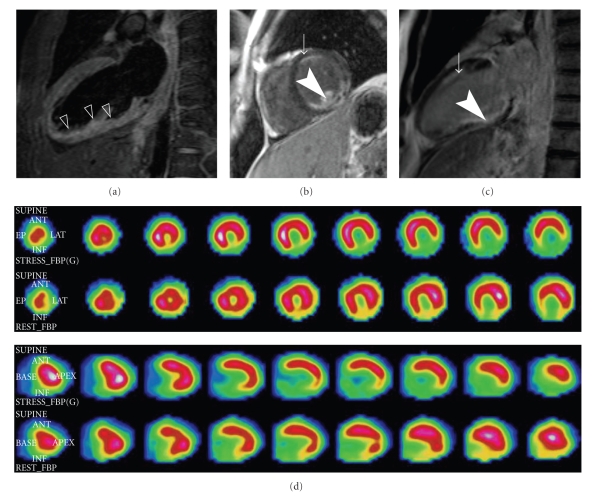
Black blood T2 images (T2 weighted short inversion-time, inversion-recovery (STIR) breath hold pulse sequences) suggested myocardial oedema (arrowheads) strictly in the inferior wall (Figure 2, Panel (e)). Gadolinium-enhanced images (Inversion Recovery turboFLASH 3D short axis and long axis sequences) demonstrated transmural late enhancement of the inferior wall (arrowheads) associated with late microvascular obstruction (Panel (f) and (g)). Furthermore, these sequences showed a subendocardial delayed enhancement area (arrows) which was located in the anterior wall at mid-ventricular level in favour of a limited necrosis (Panel (f) and (g)). Myocardial perfusion scintigraphy with thallium-201 suggested hypoperfusion in the inferior and lateral walls during stress but did not detect at rest any hypouptake in the anterior wall (Panel (h)).

A 41-year-old man was referred to our department with an inferior acute myocardial infarction. He was smoker with hyperlipidaemia (low-density lipoprotein cholesterol was 169 mg/dL). The time symptom onset to admission to the intensive care unit was ten hours. His 12-lead electrocardiogram revealed a normal sinus rhythm at 60 beats/min with persisting ST elevation and Q waves in the inferior leads. Emergency coronary angiography revealed an acute thrombotic occlusion of the second segment of the right coronary artery, a severe stenosis of a non-dominant and small circumflex coronary artery and diffuse plaques in all segments of the left anterior descending coronary artery (Figure 1, Panel (c) and (d)); the patient underwent angioplasty and (bare metal) stenting with a good final result (Figure 1, Panel (a) and (b)). Initial CMR was performed five days after the acute event. Steady-state free precession (SSFP) cine sequences showed normal wall motion in the anterior area and severe myocardial hypokinesia in the inferior wall. Black blood T2 images (T2 weighted short inversion-time, inversion-recovery (STIR) breath hold pulse sequences) suggested myocardial oedema in the inferior wall (Figure 2, Panel (e)). CMR detected transmural delayed enhancement in the inferior wall associated with late microvascular obstruction 10 minutes after gadolinium injection (Figure 2, Panel (f) and (g)). Surprisingly, another area of hyperenhancement which was subendocardial was found in the mid anterior wall of the left ventricle within the distribution of the left anterior descending coronary artery territory. 

The patient remained well and asymptomatic during follow-up. CMR and myocardial scintigraphy were also performed in this patient 3 months later. CMR again showed persistent areas of delayed enhancement in the inferior wall transmurally and subendocardial enhancement in the anterior wall at the mid-ventricular level. Myocardial scintigraphy suggested myocardial ischaemia in the inferior, lateral and basal walls in favour of a restenosis, but this study did not detect any myocardial necrosis in the anterior wall (Figure 2, Panel (h)). Restenosis on the right coronary artery was confirmed by coronary angiography. The left anterior descending artery remained unchanged with diffuse nonobstructive plaques.

## 3. Discussion

In a patient with acute inferior myocardial infarction, CMR was able to detect an unexpected coexisting area of necrosis in a different vascular territory. CMR findings in animals confirm that the subendocardial infarcts detected by CMR correspond to histologically defined myocardial infarcts [3]. In our case, despite absence of significative stenoses in the left descending coronary artery, CMR found an area of necrosis in the anterior wall. We cannot affirm that this anterior subendocardial infarction was not caused by coronary angiography. However, we hypothesise that the 2 myocardial infarctions seen on delayed enhanced MRI are the consequence of pan-coronary destabilisation, although the anterior infarct could very well be an old infarction that was so far not recognised. The lack of T2 hypersignal and the lack of microvascular obstruction in the anterior wall are in favour of a chronic myocardial infarction in this segment [4]. We can suppose that this area of limited necrosis is a consequence of a spontaneous atherosclerotic plaque rupture on the left descending coronary artery. These nonrecognised infarcts may be quite frequent and carry important prognostic implications, as it is known that spontaneous coronary atheromatous plaque rupture, without significant underlying stenosis during first acute coronary syndrome, healed without significant plaque modification in 50% of cases with medical therapy [5]. CMR is currently the best imaging technique to detect unexpected infarcts [6–8]. Thus, detecting these infarcts enables important additional information regarding to the risk stratification, eventually targeted therapy and carefully surveillance in these patients with high risk of cardiovascular events.

## 4. Conclusion

To conclude, the incidence of unrecognised coexistent myocardial infarcts may be underestimated and not infrequent. Of particular interest, this case highlights the possibility to detect these coexisting subendocardial infarcts using CMR. We believe that CMR has advantages, at least in patients with discrepancies between therapeutic success and missing clinical improvement.

**Consent**

Written informed consent was obtained from the patient for publication of this case report and any accompanying images. A copy of the written consent is available for review by the Editor-in-Chief of this journal.

**Competing Interests**

The authors declare they have no competing interests.

**Authors' Contributions**

EG, PC, HCG, MM Data acquisition, or analysis and interpretation, EG, ME, PC, MM Critical revision for important intellectual content, EG, ME Manuscript drafting, EG, HCG, ME, PC, MM Final approval of the manuscript.
